# Trends in mortality from alcohol, opioid, and combined alcohol and opioid poisonings by sex, educational attainment, and race and ethnicity for the United States 2000–2019

**DOI:** 10.1186/s12916-022-02590-z

**Published:** 2022-10-24

**Authors:** Charlotte Buckley, Yu Ye, William C. Kerr, Nina Mulia, Klajdi Puka, Jürgen Rehm, Charlotte Probst

**Affiliations:** 1 Department of Automatic Control and Systems Engineering, University of Sheffield, Sheffield, UK; 2 Alcohol Research Group, Public Health Institute, Emeryville, California USA; 3 Institute for Mental Health Policy Research, Centre for Addiction and Mental Health (CAMH), 33 Ursula Franklin Street, Toronto, ON M5S 2S1 Canada; 4 Campbell Family Mental Health Research Institute, Centre for Addiction and Mental Health, Toronto, Canada; 5 Dalla Lana School of Public Health & Department of Psychiatry, University of Toronto, Toronto, Canada; 6 Zentrum für Interdisziplinäre Suchtforschung der Universität Hamburg (ZIS), Universitätsklinikum Hamburg-Eppendorf, Hamburg, Germany; 7 Department of International Health Projects, Institute for Leadership and Health Management, I.M. Sechenov First Moscow State Medical University, Moscow, Russian Federation; 8 Heidelberg Institute of Global Health (HIGH), Medical Faculty and University Hospital, Heidelberg University, Heidelberg, Germany; 9 Department of Psychiatry, University of Toronto, Toronto, ON Canada

**Keywords:** Alcohol poisoning, Opioid poisoning, Mortality, Socioeconomic inequalities, Racial and ethnic inequalities

## Abstract

**Background:**

The ongoing opioid epidemic and increases in alcohol-related mortality are key public health concerns in the USA, with well-documented inequalities in the degree to which groups with low and high education are affected. This study aimed to quantify disparities over time between educational and racial and ethnic groups in sex-specific mortality rates for opioid, alcohol, and combined alcohol and opioid poisonings in the USA.

**Methods:**

The 2000–2019 Multiple Cause of Death Files from the National Vital Statistics System (NVSS) were used alongside population counts from the Current Population Survey 2000–2019. Alcohol, opioid, and combined alcohol and opioid poisonings were assigned using ICD-10 codes. Sex-stratified generalized least square regression models quantified differences between educational and racial and ethnic groups and changes in educational inequalities over time.

**Results:**

Between 2000 and 2019, there was a 6.4-fold increase in opioid poisoning deaths, a 4.6-fold increase in combined alcohol and opioid poisoning deaths, and a 2.1-fold increase in alcohol poisoning deaths. Educational inequalities were observed for all poisoning outcomes, increasing over time for opioid-only and combined alcohol and opioid mortality. For non-Hispanic White Americans, the largest educational inequalities were observed for opioid poisonings and rates were 7.5 (men) and 7.2 (women) times higher in low compared to high education groups. Combined alcohol and opioid poisonings had larger educational inequalities for non-Hispanic Black men and women (relative to non-Hispanic White), with rates 8.9 (men) and 10.9 (women) times higher in low compared to high education groups.

**Conclusions:**

For all types of poisoning, our analysis indicates wide and increasing gaps between those with low and high education with the largest inequalities observed for opioid-involved poisonings for non-Hispanic Black and White men and women. This study highlights population sub-groups such as individuals with low education who may be at the highest risk of increasing mortality from combined alcohol and opioid poisonings. Thereby the findings are crucial for the development of targeted public health interventions to reduce poisoning mortality and the socioeconomic inequalities related to it.

**Supplementary Information:**

The online version contains supplementary material available at 10.1186/s12916-022-02590-z.

## Background

Injury deaths, which include poisonings, are the third leading cause of death in the United States (US), after heart disease and cancer [1]. In 2019, poisonings accounted for 75,795 deaths in the US, making up 30.8% of injury deaths [1]. This increase is thought to be primarily driven by increases in opioid poisonings, and in 2019, there were 49,860 opioid-related deaths in the US, representing approximately 70% of the total poisonings [2–4]. Alcohol poisonings also represent a significant proportion of unintentional injuries and alone or in combination with other drugs caused 12,954 deaths in 2017 [5]. Poisonings overall significantly increased from 12,186 poisoning deaths in 1999 [6, 7]. Opioid and alcohol poisonings also increased substantially over this period from 8050 opioid-related and 2486 alcohol-related poisonings in 1999 [3, 5]. Alcohol is often used together with other substances, including opioids, and when used concurrently, the effects of each can be amplified [8, 9]. Thus, the risk of overdose is elevated due to sedation and respiratory depression caused by the use of opioids with alcohol. In a sample of chronic opioid users in the US, 12.4% of individuals reported concurrent alcohol use [8]. In Canada, it was estimated that in 2013, 1 in 5 fatal opioid overdoses involved alcohol [10]. In the US, alcohol was estimated to be involved in 22% of deaths associated with opioid pain relievers in 2010 [11] and 15% of opioid overdose deaths in 2017 [12].

Case and Deaton described sharp increases in “deaths of despair” between 1999 and 2013, defined as deaths from alcohol and drug poisonings, suicide, and alcoholic liver disease, affecting life expectancy among US middle-aged White males without a bachelor’s (BA) degree [13]. More recently, Case and Deaton [14, 15] argued that education is now a sharper differentiator of life expectancy than race and ethnicity. This is especially evident when comparing individuals with a BA degree, who saw life expectancy increase, to those without a BA degree, for whom life expectancy decreased since about 2010 [16]. For those with a high school degree only or less, poisoning deaths were 4 times higher in 1999, and 7.2 times higher in 2013, compared to those with a BA degree or more [14]. Richardson et al. [17] analyzed the number of US deaths from drug poisonings between 1994 and 2010 by sex, race, and educational attainment. Over this period, drug poisoning rates were highest and increased the fastest among Whites with low education.

In line with Case and Deaton’s observations, drug and alcohol poisonings appear to be increasingly related to socioeconomic status: Shiels et al. [18] also observed a gradient whereby US counties in the highest quintile of unemployment had the highest drug poisoning mortality rates and vice versa. However, the studies currently available either have not included alcohol poisoning [17] or have not decomposed poisonings by substance [14, 18] and it is unclear whether socioeconomic differences in drug and alcohol poisonings are driven by opioid, alcohol, or joint opioid and alcohol poisonings.

There is also evidence that mortality rates from drug poisonings can differ substantially by racial and ethnic group. A recent study documented disparities by race and ethnicity, with American Indian and Alaska Natives having the highest age-standardized death rates for drug poisonings, followed by non-Hispanic White and Black groups, and Hispanic and Asian groups having the lowest mortality [18].

No research to date has examined how opioids and alcohol individually and jointly contribute to socioeconomic inequalities in drug and alcohol poisonings and how these inequalities have changed over time for different racial and ethnic groups. In addition, much of the work exploring increasing socioeconomic inequalities with respect to drug and alcohol poisoning has focused on educational inequalities observed in the non-Hispanic White group, and in particular men. The aims of this study are to investigate (1) absolute disparities between (a) educational and (b) racial and ethnic groups in mortality rates for alcohol, opioid, and combined alcohol and opioid poisonings over time (2000–2019) and (2) whether relative educational inequalities in poisonings from alcohol, opioid, and combined alcohol and opioid are observed to the same extent within different race and ethnicity groups over time (2000–2019).

## Methods

### Data

Mortality data from individual death records for the years 2000–2019 were obtained from the National Vital Statistics System (NVSS) [19]. Aggregate-level Multiple Cause of Death Files were used, containing nearly complete information on education, race and ethnicity, age, and sex of the deceased (recorded at time of death) in addition to the underlying and up to 20 contributing causes of death coded according to the ICD-10. Corresponding population estimates were based on Current Population Surveys (CPS) [20].

### Measures

Sex was classified as men and women. Race and ethnicity group was determined from the Hispanic origin/race recode measure and was categorized into four groups: (1) non-Hispanic White, hereafter White; (2) non-Hispanic Black, hereafter Black; (3) Hispanic; or (4) non-Hispanic others (mixed). Education was determined from two types of reporting systems (1989 or 2003 revision) and was classified into three categories: (1) high school degree or less (12 years or less of school, less or equal to high school graduate, or GED completed), hereafter low education; (2) some college (1–3 years of college, some college credit but no degree, or associate degree), hereafter medium education; or (3) college degree or more (at least 4 years of college or bachelor’s degree or higher), hereafter high education. Complete data was available for sex and race and ethnicity. Approximately 3.8% of alcohol or opioid deaths were missing for education, which were re-assigned as low to high education based on the education distribution within a given sex-race/ethnicity-(5-year) age group for each year.

### Classification of poisoning deaths

We defined opioid and alcohol poisoning deaths using ICD-10 underlying and contributing cause-of-death and then recoded them into three non-overlapping groups: (1) alcohol-only poisoning, (2) opioid-only poisoning, or (3) both alcohol and opioid poisoning. Cause-of-death codes for alcohol and opioid poisoning classifications are displayed in Additional file 1: Table S1 and Fig. S1. Three versions of classifications were explored to define alcohol poisoning, each affecting the coding of deaths from alcohol, opioid, and combined alcohol and opioid poisoning (see Additional file 1 for details). Opioid poisoning must meet both of the following criteria: (1) X40-X44 (unintentional poisoning), X60-X64 (suicide, i.e., intentional self-poisoning), X85 (homicide, i.e., assault by drug medicaments and biological substances), or Y10-Y14 (poisoning with undetermined intent) from underlying cause with drug overdose among deaths and (2) T40.0-T40.4 or T40.6 (opioid) from contributing cause [21, 22].

Our main analysis focused on individuals aged over 25 years as education may still be ongoing before that age, and to be consistent with the US Census Bureau, with sensitivity analyses conducted including all individuals aged 18 and older [23]. Additional file 1: Table S2 shows the number of deaths from alcohol-only poisoning, opioid-only poisoning, and alcohol and opioid poisoning for the total US population, age 18 and older and age 25 and older, separately. The number of cause-specific deaths dropped by a maximum of 10% when restricting the age range to 25 and older. All mortality rates were age-standardized using population age distributions for 5-year age groups for the year 2019 CPS data and expressed as deaths per 100,000 population.

### Statistical analysis

Sex-specific trends (2000–2019) in US mortality rates on the three poisoning outcomes (alcohol-only, opioid-only, and alcohol and opioid) by education (with and without stratification by race and ethnicity) are presented. Generalized least square (GLS) regression models were fit to estimate differences between educational and race and ethnicity groups. Modeling results aim to provide estimates on the poisoning mortality rate over time for (1) independent effects of education and race and ethnicity groups (objective 1) and (2) differential education effects by race and ethnicity (objective 2).

Two types of GLS models were fit to answer the two research questions. In the first analysis (objective 1), the effects of education and race and ethnicity on mortality over time were quantified for all three poisoning outcomes. For each sex, a panel was constructed with age-standardized mortality rates (in 100,000 population) split by education and race and ethnicity category for each year, i.e., *n* = 240 for each model (20 years × 4 race/ethnic groups × 3 education groups). The GLS model allows for heteroskedastic variances and panel-specific, first-order auto-regression. The three poisoning outcomes were fit in separate models. Predictors were education and race and ethnicity dummy variables, linear year, year squared, and the interactions between linear and squared year and education and between linear and squared year and race and ethnicity. Both year measures were centered at 2010; thus, the main effects from education and race and ethnicity estimate the average absolute difference in mortality rates across education and race and ethnicity groups in 2010. Year squared was included to capture the non-linear increase that has been observed in poisoning mortality rates over the time period. Interaction effects estimate average differences in changes in mortality rate over time across groups. Several sensitivity analyses were performed to check the robustness of the results. Both analyses were repeated with all individuals aged 18 and older included. As an alternative to GLS modeling, Poisson random effect models were estimated on mortality death counts with population as an offset.

The first analysis assumed no interaction between education, race and ethnicity, and time. In the second analysis (objective 2), this assumption is relaxed and the relative differential effects of education across race and ethnicity groups were quantified. Mortality rate ratios were calculated by dividing mortality rates for individuals with low or medium education by mortality rates for individuals with high education. Using GLS models, the mortality rate ratios were regressed on race and ethnicity dummy variables, the linear year (centered at 2010), and the interaction between year and race and ethnicity. The main effects of race and ethnicity estimate the average differential education effects by race and ethnicity in the year 2010, while the interactions indicate whether and how educational inequalities in poisoning deaths changed over time in different race and ethnicity groups.

## Results

In individuals aged 25 and older, there were 3747 alcohol-only, 5997 opioid-only, and 1641 combined alcohol and opioid poisoning deaths in 2000, compared with 7742 alcohol-only, 38544 opioid-only, and 7497 combined alcohol and opioid poisoning deaths in 2019 (Additional file 1: Table S2). This represented a 2.1-fold increase in alcohol-only poisonings, a 6.4-fold increase in opioid-only poisonings, and a 4.6-fold increase in combined alcohol and opioid poisonings. Opioid-only poisonings represented 53% (2000) and 72% (2019) of the total poisonings studied while alcohol-only represented 33% (2000) and 14% (2019) and combined alcohol and opioid represented 14% (2000) and 14% (2019).

**Fig. 1:**
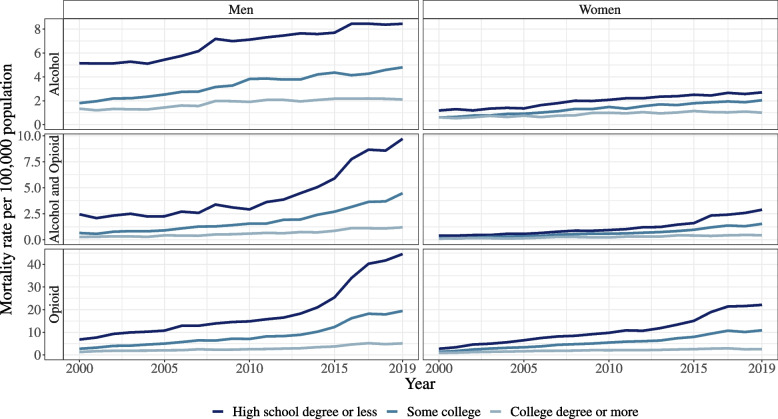
Age-standardized mortality rates for alcohol-only poisoning, opioid-only poisoning, and combined alcohol and opioid poisoning for men and women by educational attainment categories from 2000 to 2018. Note: *Alcohol-only poisoning*: ICD-10 code X45 or F10.0 from underlying or contributing cause or T51.0 or T51.9 from contributing cause, and *not* opioid poisoning, i.e., both (**a**) X40-X44, X60-X64, X85, or Y10-Y14 from underlying cause and (**b**) T40.0-T40.4 or T40.6 from contributing cause. *Opioid-only poisoning*: both (**a**) X40-X44, X60-X64, X85, or Y10-Y14 from underlying cause and (**b**) T40.0-T40.4 or T40.6 from contributing cause, and *not* alcohol poisoning, i.e., X45 or F10.0 from underlying or contributing cause or T51.0 or T51.9 from contributing cause. *Alcohol and opioid poisoning*: alcohol poisoning, i.e., X45 or F10.0 from underlying or contributing cause or T51.0 or T51.9 from contributing cause, *and* opioid poisoning, i.e., both (**a**) X40-X44, X60-X64, X85, or Y10-Y14 from underlying cause and (**b**) T40.0-T40.4 or T40.6 from contributing cause

Men with low education had an age-standardized mortality rate of 5.2 (2000) and 8.4 (2019) per 100,000 population for alcohol-only, 6.8 (2000) and 44.6 (2019) for opioid-only, and 2.5 (2000) and 9.7 (2019) for combined alcohol and opioid poisonings. By contrast, the age-standardized mortality rate for men with high education was 1.3 (2000) and 2.1 (2019) for alcohol-only, 1.3 (2000) and 5.1 (2019) for opioid-only, and 0.3 (2000) and 1.2 (2019) for combined alcohol and opioid poisonings. For women with low education, the age-standardized mortality rate was 1.2 (2000) and 2.7 (2019) for alcohol-only, 2.7 (2000) and 22.1 (2019) for opioid-only, and 0.4 (2000) and 2.9 (2019) for alcohol and opioid poisonings. Women with high education had lower corresponding mortality rates of 0.6 (2000) and 1.0 (2019) for alcohol-only, 0.9 (2000) and 2.6 (2019) for opioid-only, and 0.1 (2000) and 0.4 (2019) for alcohol and opioid poisonings (Fig. 1).

Text comparisons below will focus on inequalities between low and high education groups, with comparisons between medium and high education groups summarized in Tables 1 and 2. For all sensitivity analyses, results were broadly consistent with the main analyses (Additional file 1: Tables S3, S4 and S5).

**Table 1:** Coefficient estimates of generalized least square (GLS) models predicting racial and ethnic and educational differences in US poisoning mortality rates (per 100,000) aged 25 and older 2000–2019^a^

	Men			Women		
	Alcohol-only	Opioid-only	Alcohol and opioid	Alcohol-only	Opioid-only	Alcohol and opioid
Race and ethnicity effect at 2010 (ref. White)						
Black	−1.017 (−1.274, −0.761)^***^	−4.542 (−9.279, 0.196)	−0.694 (−1.459, 0.071)	−1.024 (−1.236, −0.813)^***^	−4.189 (−7.515, −0.864)^*^	−0.512 (−0.779, −0.245)^***^
Hispanic	−1.339 (−1.849, −0.829)^***^	−5.394 (−11.115, 0.328)	−0.804 (−1.570, −0.037)^*^	−0.902 (−1.077, −0.728)^***^	−7.985 (−13.285, −2.685)^**^	−0.451 (−0.614, −0.288)^***^
Other	−0.005 (−0.362, 0.353)	−5.837 (−9.968, −1.706)^**^	−1.143 (−1.668, −0.618)^***^	−0.565 (−0.745, −0.384)^***^	−4.790 (−8.014, −1.566)^**^	−0.455 (−0.605, −0.306)^***^
Education effect at 2010 (ref high education)						
Low education	5.554 (5.208, 5.901)^***^	21.839 (11.679, 32.000)^***^	3.109 (1.414, 4.804)^***^	1.158 (0.924, 1.393)^***^	6.344 (4.473, 8.215)^***^	0.043 (−0.604, 0.690)
Medium education	1.581 (1.364, 1.797)^***^	3.678 (1.299, 6.057)^**^	0.870 (−0.131, 1.871)	0.230 (0.010, 0.450)^*^	1.946 (1.071, 2.820)^***^	−0.196 (−0.807, 0.415)
Linear year^b^	0.068 (0.052, 0.083)^***^	0.486 (0.032, 0.941)^*^	0.087 (0.001, 0.173)^*^	0.043 (0.021, 0.066)^***^	0.427 (0.056, 0.799)^*^	0.097 (0.034, 0.160)^**^
Quadratic year^b^	−0.003 (−0.006, 0.000)^*^	0.016 (−0.009, 0.040)	0.002 (−0.003, 0.007)	−0.002 (−0.005, 0.001)	0.010 (−0.009, 0.029)	0.003 (0.000, 0.006)
Race by linear year						
Black × year	−0.052 (−0.081, −0.023)^***^	−0.280 (−0.716, 0.157)	0.026 (−0.049, 0.102)	−0.034 (−0.055, −0.013)^**^	−0.353 (−0.717, 0.012)	−0.017 (−0.045, 0.010)
Hispanic × year	−0.068 (−0.123, −0.013)^*^	−0.717 (−1.207, −0.227)^**^	−0.106 (−0.180, −0.032)^**^	−0.059 (−0.080, −0.039)^***^	−0.920 (−1.572, −0.268)^**^	−0.064 (−0.083, −0.045)^***^
Other × year	−0.010 (−0.054, 0.033)	−0.699 (−1.149, −0.248)^**^	−0.159 (−0.215, −0.102)^***^	−0.024 (−0.045, −0.003)^*^	−0.491 (−0.872, −0.110)^*^	−0.070 (−0.088, −0.052)^***^
Race by quadratic year						
Black × year sq.	0.007 (0.002, 0.012)^**^	0.019 (−0.013, 0.050)	0.012 (0.003, 0.020)^**^	0.004 (0.001, 0.007)^**^	−0.003 (−0.023, 0.017)	0.002 (−0.001, 0.005)
Hispanic × year sq.	0.004 (−0.003, 0.012)	−0.030 (−0.061, 0.001)	0.000 (−0.007, 0.008)	0.001 (−0.002, 0.005)	−0.037 (−0.071, −0.004)^*^	−0.003 (−0.006, 0.000)^*^
Other × year sq.	0.003 (−0.004, 0.010)	−0.029 (−0.055, −0.002)^*^	−0.005 (−0.012, 0.001)	0.002 (−0.001, 0.006)	−0.016 (−0.037, 0.004)	−0.003 (−0.005, 0.000)^*^
Education by linear year						
Low education × year	0.186 (0.148, 0.225)^***^	2.132 (1.450, 2.814)^***^	0.322 (0.184, 0.460)^***^	0.073 (0.049, 0.097)^***^	0.809 (0.619, 0.999)^***^	0.032 (−0.037, 0.101)
Med education × year	0.108 (0.083, 0.132)^***^	0.609 (0.366, 0.852)^***^	0.144 (0.040, 0.247)^**^	0.053 (0.031, 0.075)^***^	0.255 (0.168, 0.343)^***^	−0.006 (−0.069, 0.056)
Educ by quadratic year						
Low education × year sq.	0.004 (−0.002, 0.011)	0.122 (0.085, 0.159)^***^	0.026 (0.014, 0.038)^***^	0.003 (−0.001, 0.006)	0.039 (0.024, 0.055)^***^	0.005 (0.001, 0.009)^*^
Med education × year sq.	0.000 (−0.004, 0.005)	0.042 (0.019, 0.065)^***^	0.011 (0.004, 0.017)^**^	0.001 (−0.002, 0.005)	0.012 (0.001, 0.022)^*^	0.001 (−0.003, 0.004)
Intercept^c^	2.155 (2.017, 2.293)^***^	5.217 (1.032, 9.401)^*^	0.974 (−0.203, 2.150)	1.413 (1.190, 1.637)^***^	5.008 (1.869, 8.146)^**^	0.956 (0.339, 1.572)^**^

^*^p<.05

^**^p<.01

^***^p<.001

^a^Model predictors are linear and quadratic year centered at 2010, race and ethnicity and education dummies, and interaction between year, year square, and race/ethnicity, and between year, year square, and education. *N*=240 for each model (20 years × 4 race groups × 3 education groups). All models allow for heteroskedastic variances and panel-specific first-order auto-regression (panel defined by race by education combination)

^b^Linear and quadratic year effect for the reference group, i.e., white with high education

^c^Intercept estimates average mortality rate for white with high education at year 2010

**Table 2:** Coefficient estimates of generalized least square (GLS) models predicting racial and ethnic differences in educational inequalities in US poisoning mortality ratios calculated from mortality rates (per 100,000) aged 25 and older 2000–2019^a^

	Alcohol-only		Opioid-only		Alcohol and opioid	
**Men**	**Low education to high**	**Med education to high**	**Low education to high**	**Med education to high**	**Low education to high**	**Med education to high**
Race effect at 2010 (ref. White)						
Black	1.48 (0.96, 2.01)^***^	0.29 (0.04, 0.53)^*^	−0.55 (−1.74, 0.64)	−0.08 (−0.48, 0.32)	1.50 (0.52, 2.47)^**^	0.36 (−0.04, 0.76)
Hispanic	0.48 (−0.24, 1.20)	0.38 (0.06, 0.69)^*^	−2.55 (−3.54, −1.56)^***^	−0.06 (−0.57, 0.46)	−1.74 (−2.75, −0.73)^**^	0.40 (−0.07, 0.86)
Other	8.27 (7.43, 9.10)^***^	3.04 (2.65, 3.44)^***^	1.62 (−0.16, 3.41)	1.58 (0.51, 2.65)^**^	9.23 (1.98, 16.49)^*^	3.94 (1.71, 6.18)^**^
Year effect^b^	−0.01 (−0.02, 0.00)^*^	0.03 (0.02, 0.04)^***^	0.26 (0.22, 0.29)^***^	0.10 (0.09, 0.11)^***^	0.00 (−0.09, 0.08)	0.05 (0.03, 0.07)^***^
Race by year						
Black	0.09 (0.00, 0.18)^*^	0.01 (−0.03, 0.06)	0.04 (−0.15, 0.24)	0.02 (−0.05, 0.09)	0.37 (0.20, 0.54)^***^	0.11 (0.04, 0.18)^**^
Hispanic	−0.04 (−0.17, 0.08)	−0.02 (−0.07, 0.04)	−0.23 (−0.40, −0.06)^**^	−0.04 (−0.13, 0.04)	0.10 (−0.07, 0.27)	0.05 (−0.03, 0.13)
Other	0.09 (−0.06, 0.24)	0.11 (0.04, 0.18)^**^	−0.18 (−0.49, 0.12)	−0.03 (−0.21, 0.15)	−0.43 (−1.67, 0.81)	−0.26 (−0.65, 0.13)
Intercept^c^	3.80 (3.73, 3.86)^***^	1.79 (1.74, 1.84)^***^	7.54 (7.31, 7.77)^***^	3.00 (2.95, 3.05)^***^	7.40 (6.91, 7.88)^***^	2.76 (2.65, 2.87)^***^
**Women**	**Low education to high**	**Med education to high**	**Low education to high**	**Med education to high**	**Low education to high**	**Med education to high**
Race effect at 2010 (ref. White)						
Black	1.25 (0.54, 1.96)^**^	0.17 (−0.19, 0.53)	−1.79 (−3.64, 0.06)	−0.64 (−0.99, −0.28)^***^	6.00 (2.63, 9.37)^***^	1.35 (0.17, 2.53)^*^
Hispanic	−1.12 (−1.43, −0.80)^***^	−0.07 (−0.32, 0.17)	−4.19 (−5.28, −3.10)^***^	−0.48 (−1.11, 0.15)	−2.20 (−2.91, −1.50)^***^	0.02 (−0.93, 0.97)
Other	7.09 (5.48, 8.70)^***^	3.16 (2.16, 4.16)^***^	−0.02 (−1.30, 1.26)	1.68 (1.01, 2.34)^***^	7.69 (−3.48, 18.87)	4.08 (0.75, 7.42)^*^
Year effect^b^	0.04 (0.02, 0.06)^***^	0.04 (0.03, 0.05)^***^	0.43 (0.33, 0.54)^***^	0.15 (0.12, 0.18)^***^	0.19 (0.11, 0.27)^***^	0.10 (0.06, 0.14)^***^
Race by year						
Black	−0.04 (−0.17, 0.08)	−0.04 (−0.10, 0.02)	−0.11 (−0.37, 0.15)	−0.03 (−0.08, 0.03)	−0.27 (−0.84, 0.30)	−0.08 (−0.28, 0.12)
Hispanic	0.05 (−0.01, 0.10)	0.06 (0.02, 0.10)^**^	−0.50 (−0.66, −0.33)^***^	−0.18 (−0.29, −0.07)^**^	−0.05 (−0.18, 0.07)	0.03 (−0.14, 0.19)
Other	−0.30 (−0.58, −0.02)^*^	−0.02 (−0.19, 0.16)	−0.25 (−0.45, −0.05)^*^	−0.05 (−0.17, 0.06)	1.45 (−0.45, 3.36)	0.46 (−0.12, 1.05)
Intercept^c^	2.58 (2.48, 2.68)^***^	1.55 (1.49, 1.61)^***^	7.22 (6.41, 8.04)^***^	3.00 (2.77, 3.23)^***^	4.85 (4.39, 5.31)^***^	2.32 (2.08, 2.56)^***^

^*^p<.05

^**^p<.01

^***^p<.001

^a^Model predictors are linear year centered at 2010, race and ethnicity, and interaction between year and race and ethnicity. *N*=80 for each model (20 years × 4 race groups) except for models predicting alcohol and poisoning with *N*=79 for men and *N*=62 for women with missing years having zero mortality rate for high education. All models allow for heteroskedastic variances and panel-specific first-order auto-regression (panel defined by race and ethnicity). Model outcomes are low and medium education mortality rate divided by high education mortality rate, separately

^b^Linear year effect for the reference group, i.e., white

^c^Intercept estimates average mortality education ratio for white at year 2010

### Objective 1: Educational and racial and ethnic differences in poisoning mortality

Educational inequalities were observed for most poisoning outcomes and these inequalities were largest for opioid-only poisonings for both men and women, finding that low education (relative to high education) was associated with 21.8 (men) and 6.3 (women) additional deaths per 100,000 persons in 2010. For alcohol-only poisonings, those with low education had 5.6 (men) and 1.2 (women) additional deaths per 100,000. For combined alcohol and opioid poisonings, mortality rates in 2010 were higher by 3.1 (men) and 0.04 (women, not significant) deaths per 100,000 for low compared to high education groups. Positive interactions between education and linear year were observed for most poisoning outcomes and the positive interactions between education and quadratic year were observed for opioid only and combined alcohol and opioid, indicating that not only the absolute educational inequality in poisoning mortality rates widened over time, but also the inequality accelerated in more recent years particularly for opioid poisoning.

Differences in poisoning death rates between race and ethnicity groups were observed with some interaction effects between linear and quadratic year and race and ethnicity group (Table 1), consistent with the trend in Additional file 1: Fig. S2 (not stratified by educational attainment). The results largely show that in 2010, White men and women had the poorest outcomes for all poisoning types. Poisoning mortality rates for Hispanic individuals did not increase as fast as Whites did over time. However, some positive interaction effects for Black men and women indicate the racial and ethnic differences in 2010 diminished or even reversed over time in some cases. For example, Black men’s combined alcohol and opioid death rates accelerated and surpassed White men’s rates by 2019 (Additional file 1: Fig. S2).

### Objective 2: Educational inequalities within race and ethnicity groups in poisoning mortality

**Fig. 2:**
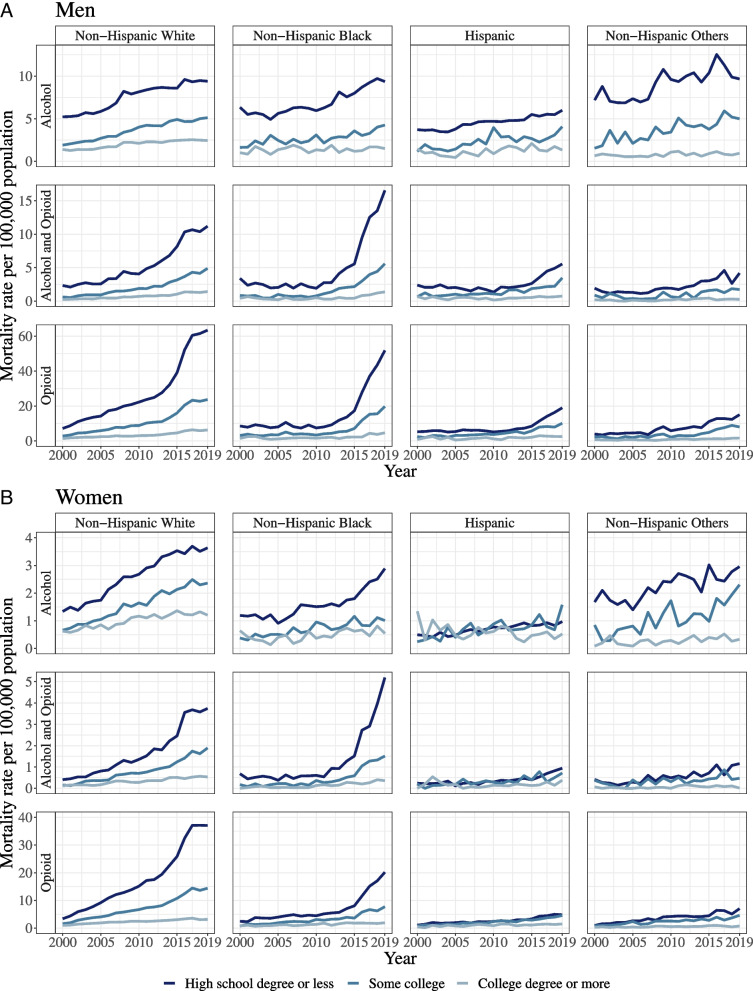
Mortality per 100,000 alcohol-only, opioid-only, and alcohol and opioid poisoning for men and women aged 25 and oldere split by educational attainment and race and ethnicity categories for 2000–2018. Note: *Alcohol-only poisoning*: ICD-10 code X45 or F10.0 from underlying or contributing cause or T51.0 or T51.9 from contributing cause, and *not* opioid poisoning, i.e., both (**a**) X40-X44, X60-X64, X85, or Y10-Y14 from underlying cause and (**b**) T40.0-T40.4 or T40.6 from contributing cause. *Opioid-only poisoning*: both (**a**) X40-X44, X60-X64, X85, or Y10-Y14 from underlying cause and (**b**) T40.0-T40.4 or T40.6 from contributing cause, and *not* alcohol poisoning, i.e., X45 or F10.0 from underlying or contributing cause or T51.0 or T51.9 from contributing cause. *Alcohol and opioid poisoning*: alcohol poisoning, i.e., X45 or F10.0 from underlying or contributing cause or T51.0 or T51.9 from contributing cause, *and* opioid poisoning, i.e., both (**a**) X40-X44, X60-X64, X85, or Y10-Y14 from underlying cause and (**b**) T40.0-T40.4 or T40.6 from contributing cause

Figure 2 summarizes mortality rates over time (2000–2019) from alcohol-only, opioid-only, and combined alcohol and opioid poisonings split by educational attainment and race and ethnicity. As shown in Table 2, for Whites, the largest relative educational differences were observed for opioid-only poisonings with 7.5 (men) and 7.2 (women) times more opioid poisonings in those with low education compared to high education. There were large inequalities in combined alcohol and opioid poisonings, particularly for men with a mortality rate 7.4 times higher in low education groups. For women’s combined alcohol and opioid poisonings, educational inequalities were smaller but substantial (4.9 times higher in low compared to high education). The smallest inequalities were observed for alcohol-only poisonings, with mortality rates being 3.8 (men) and 2.6 (women) times higher. A significant positive effect of year was observed for all poisoning types for White women and for opioid-only poisoning for White men, indicating widening relative educational inequalities in each year.

The educational inequality in mortality rates (comparing low with high education) for Black men (relative to White) was 1.5 (both alcohol-only and combined alcohol and opioids) times greater in 2010, but with no difference observed for opioid-only poisonings. This resulted in mortality rates for Black men that were 5.3 (alcohol-only) and 8.9 (combined alcohol and opioid) times higher in low compared to high education groups. A significant interaction between time and race and ethnicity for combined alcohol and opioid poisonings indicated that educational inequalities have increased more for Black men compared to White men over time. For Hispanic men and women, the trend for opioid-only poisonings followed the opposite pattern, indicating that educational inequalities increased less for Hispanic compared to White men and women. Among Black women, educational inequalities for combined alcohol and opioid mortality rates were larger by 6.0 compared to White women, resulting in mortality rates 10.9 times higher in Black women with low compared to high education. For women, there was no interaction effect between time and race and ethnicity, indicating that widening inequalities over time were similar to Whites.

## Discussion

This study provides for the first time a detailed overview of poisoning mortality from alcohol-only, opioid-only, and combined alcohol and opioids by educational attainment and race and ethnicity in US men and women. Large and increasing educational inequalities were found between those with and without a college degree in alcohol-only, opioid-only, and combined alcohol and opioid poisonings, with particularly high mortality in those with a high school degree or less. The relative educational inequalities in alcohol-only, opioid-only, and alcohol and opioid poisonings between racial and ethnic groups over time were also quantified. We find that educational inequalities in poisoning deaths were most pronounced in non-Hispanic White and Black men and women. It appears that these socioeconomic differences in drug and alcohol poisonings have been primarily driven by opioid-only poisonings, which caused 53% of the poisonings studied in 2000, rising to 72% in 2018. Despite a dramatic increase in opioid poisonings, the proportion of poisonings caused by combined alcohol and opioids remained stable.

These findings support previous work by Case and Deaton, which found large inequalities between non-Hispanic White individuals with and without a BA degree in poisoning mortality [13]. They also support more recent findings by these authors that suggest that while gaps in mortality between race and ethnic groups have decreased, inequalities in mortality between educational groups have increased. Our results build on this work and suggest that for poisoning deaths, these widening educational inequalities are occurring both in the adult population overall and within racial and ethnic groups. We have shown growing inequality between educational categories for non-Hispanic Black and White groups for all types of poisoning deaths considered and that the inequalities have grown dramatically between those with a high school degree or less compared to those with a college degree. Additionally, our results suggest that relative educational inequalities in combined alcohol and opioid poisoning mortality may be the largest and increasing the most over time for non-Hispanic Black individuals. Consistent with this, a recent report by the US Substance Abuse and Mental Health Services Administration (SAMHSA) highlights dramatic growth in opioid overdose deaths in Black communities [24]. A number of factors could be driving this, including increased availability of pure heroin, greater presence of potent synthetic opioids such as fentanyl in illicit drug markets, and racial and ethnic and neighborhood disparities in access to medications for treating opioid use disorders [24, 25].

In addition to providing a detailed overview of the poisonings from different substances by race and ethnicity and education, we provide a new method for categorizing both alcohol and combined alcohol and opioid poisonings. This method avoids previous issues noted with the changing of ICD-10 codes F10.0 that has been previously documented [26]. This provides a new methodology for capturing trends in alcohol poisonings that avoids jumps in the data.

### Limitations

We were unable to separate US-born and non-US-born Hispanic individuals; however, there may be key differences between these populations. Prior research shows that non-US-born Hispanic individuals are less likely to use substances and more likely to have lower educational attainment [27, 28], and thus aggregating across these groups could potentially obscure important patterns and trends.

One further limitation is that we were not able to consider a breakdown of the rather heterogeneous, non-Hispanic others group including mixed race and ethnicity, due to the small numbers of poisonings observed in some sub-categories (e.g., alcohol poisonings in those with a BA degree). Although we were unable to draw conclusions about this group from our data, our results for the non-Hispanic other race and ethnicity group are similar to previous findings, specifically, differences in unintentional injury mortality (including poisonings) in Native American and Alaska Native individuals, with these individuals having a rate eight times higher than non-Hispanic Whites for alcohol poisoning [26]. Since these individuals are more likely to be in the high school degree or less educational category [29], this could explain the disparities in the present study in alcohol-only poisonings between non-Hispanic others with low and high education.

While we did not consider substances other than alcohol and opioids, there are other substances that may be contributing to the pooled drug and alcohol poisonings [13]. Specifically, recent data suggests that a “fourth wave” of the opioid epidemic may have been entered, characterized by substantial co-involvement of opioid poisonings with cocaine, amphetamine, and benzodiazepines in 2019 [30]. One substance that is important to consider in future work is benzodiazepines, which have recently been estimated to be involved in 21% of opioid poisonings. It is unclear whether there are socioeconomic inequalities in these. In this study, we were only able to explore differences by educational attainment and race and ethnicity. Future work should consider other demographic factors, including additional facets of socioeconomic status, specific age groups, and urban vs. rural locations [31]. The results of this study are only applicable to a US context and would not be generalizable to other countries with differing socioeconomic inequalities and access to healthcare. Although our method for defining alcohol poisonings avoids previously documented inconsistencies in the data, this method is yet to be validated by an external expert committee. Finally, there are potential biases in the coding of poisonings, which could lead to an underestimation of the number of opioid poisonings presented in the current analysis [32]. It is also possible that due to stigma, these biases are unequally distributed across groups, for example, individuals with higher socioeconomic status may be less likely to be assigned an opioid poisoning category. It is important to consider in future work how these biases may relate to the under-recording of opioid-only and combined opioid poisonings and how these differ across sociodemographic groups.

Our findings demonstrate the increasing concentration of poisoning deaths among individuals with low socioeconomic status. This may be indicative of specific developments in the opioid crisis as well as societal trends of growing despair. In more recent years, a shift in the opioid crisis has been observed with declining opioid prescription rates, increases in poisoning deaths from illicitly manufactured opioids, and greater presence of opioids mixed with toxic adulterants [33]. This has coincided with increasing exposure to more potent substances such as cheaper synthetic opioids like fentanyl in illicit drug markets. Combined with disparities in access to effective substance use treatment [24, 25], these developments might be driving the increasing concentration of poisoning deaths in lower SES and Black populations we observed. These underlying dynamics of the opioid crisis might explain an increase in opioid-only and combined alcohol and opioid poisonings in lower socioeconomic groups. However, we also find rising alcohol-only poisonings in these groups, which cannot be explained by these mechanisms alone and may instead be a symptom of wider despair in society [15, 34]. Large parts of society at the lower end of the socioeconomic spectrum have experienced increasing levels of economic hardship, job insecurity, uncertainty about the future, and disruptions in the social fabric of their communities [15, 34]. Public health strategies should focus on intervening through a combination of these mechanisms. These include harm reduction strategies such as the provision of safer drug use spaces and expanded access to naloxone [35], equitable access to effective treatments for opioid use disorders, and a wider health-in-all-policies approach that encompasses policies on affordable or universal health care and strengthening of social welfare systems to decrease despair. Our finding that those with low education have dramatically rising poisoning death rates is also important to consider in the context of educational attainment patterns by race and ethnicity. In 2017, nearly half (46%) of Black Americans aged 25 or older had only a high school degree or less compared to one-third (34%) of White Americans [36] and thus might be disproportionately affected by these recent trends. Finally, the COVID-19 pandemic appears to have exacerbated mortality trends. Deaths from drug poisonings have increased further [37], especially in Black populations [38], and this trend has corresponded with increases in socioeconomic [39] and racial and ethnic [40] inequalities in mortality that are directly and indirectly related to COVID-19. Therefore, it is likely that there will be further widening of socioeconomic inequalities in poisoning mortality with differences across US racial and ethnic groups.

## Conclusions

Educational inequalities are present in alcohol-only, opioid-only, and combined alcohol and opioid poisonings and have increased substantially between 2000 and 2019, particularly for non-Hispanic Black and White groups. Opioid poisonings continue to represent the largest proportion of poisonings and have the highest inequalities in mortality between those with low and high education. Quantifying these differences highlights where interventions should target to aim to reduce harms from poisonings in the USA, including a focus on groups with low education. Future research is needed to understand drivers of these inequalities to inform targeted interventions to reduce poisoning mortality rates and socioeconomic inequalities.

## Supplementary Information

**Additional file 1: Fig. S1.** Total number of deaths aged 18 or older in US 2000-19 from alcohol poisoning only, opioid poisoning only and alcohol and opioid poisoning using three different versions of alcohol poisoning definition. **Fig. S2.** Age-standardized mortality rates for alcohol poisoning, opioid poisoning and combined alcohol and opioid poisoning for men and women by race and ethnicity categories from 2000 to 2019. **Table S1.** ICD-10 Codes used to define alcohol and opioid poisoning cause-of-death. **Table S2.** Number of deaths by three versions of alcohol poisoning definition (raw, adjusted and final) for alcohol poisoning only, opioid poisoning only and alcohol and opioid poisoning, for the total population, age 18 or older, and age 25 or older. **Table S3.** Coefficient estimates of generalized least square (GLS) models predicting racial and ethnic and educational differences in US poisoning mortality rates (per 100,000) aged 18 and over 2000-2019. **Table S4.** Coefficient estimates of generalized least square (GLS) models predicting racial and ethnic differences in educational inequalities in US poisoning mortality ratios calculated from mortality rates (per 100,000) aged 18 and more 2000-2019. **Table S5.** Coefficient estimates of random-effect Poisson models predicting racial and ethnic and educational differences in US poisoning death counts aged 25 and more 2000-2019.

## Data Availability

The data that support the findings of this study are publicly available from the National Vital Statistics System [https://www.cdc.gov/nchs/data_access/vitalstatsonline.htm#Mortality_Multiple] and Current Population Surveys [https://www.census.gov/programs-surveys/cps/data/datasets.html].

## Abbreviations

BA: Bachelor’s
CPS: Current Population Surveys
GLS: Generalized least squares
NVSS: National Vital Statistics System
US: United States
