# Supplementary material for: Trends in mortality from alcohol, opioid, and combined alcohol and opioid poisonings by sex, educational attainment, and race and ethnicity for the United States 2000–2019
Source: BMC Med. 2022 Oct 24;20:405. doi: 10.1186/s12916-022-02590-z (PMC9590383; doi:10.1186/s12916-022-02590-z)
Supplement: Supplementary file 1 — Additional file 1: Fig. S1. Total number of deaths aged 18 or older in US 2000-19 from alcohol poisoning only, opioid poisoning only and alcohol and opioid poisoning using three different versions of alcohol poisoning definition. Fig. S2. Age-standardized mortality rates for alcohol poisoning, opioid poisoning and combined alcohol and opioid poisoning for men and women by race and ethnicity categories from 2000 to 2019. Table S1. ICD-10 Codes used to define alcohol and opioid poisoning cause-of-death. Table S2. Number of deaths by three versions of alcohol poisoning definition (raw, adjusted and final) for alcohol poisoning only, opioid poisoning only and alcohol and opioid poisoning, for the total population, age 18 or older, and age 25 or older. Table S3. Coefficient estimates of generalized least square (GLS) models predicting racial and ethnic and educational differences in US poisoning mortality rates (per 100,000) aged 18 and over 2000-2019. Table S4. Coefficient estimates of generalized least square (GLS) models predicting racial and ethnic differences in educational inequalities in US poisoning mortality ratios calculated from mortality rates (per 100,000) aged 18 and more 2000-2019. Table S5. Coefficient estimates of random-effect Poisson models predicting racial and ethnic and educational differences in US poisoning death counts aged 25 and more 2000-2019. [file 12916_2022_2590_MOESM1_ESM.docx]

Additional File 1

Table S1. ICD-10 Codes used to define alcohol and opioid poisoning cause-of-death

| Cause-of-death | ICD-10 Codes |
| --- | --- |
| Opioid poisoning | 1. X40-X44, X60-X64, X85, Y10-Y14 from underlying cause 2. T40.0-T40.4, T40.6 from contributing cause   Must meet both 1) and 2) |
| Alcohol poisoning |  |
| Version 1 (Raw) | X45 (underlying or contributing cause) |
| Version 2 (Adjusted) | X45 or F10.0 (underlying or contributing cause) |
| Version 3 (Final) | X45 or F10.0 (underlying or contributing cause), T51.0 or T51.9 (contributing cause) |

Three versions of classifications were explored to define alcohol poisoning, each affecting the coding of deaths from alcohol poisoning only, opioid-only, and both alcohol and opioid poisoning. The death counts resulting from each of the versions is reported in Appendix Figure A1 and Appendix Table A2. The raw version (#1) was simply ICD-10 code X45 (Accidental poisoning by and exposure to alcohol) from either underlying or contributing cause. Under the raw version definition there is a jump in deaths from 2006 to 2007 for alcohol-only, as well as alcohol and opioid poisoning. Since ICD-10 code F10.0 (acute alcohol intoxication) was discontinued in 2007 (23), a natural choice for correcting the apparent inconsistency in ICD-10 coding for alcohol poisoning is to combine X45 and F10.0 (here, both underlying and contributing cause are used). Under this adjusted version (#2), the trends in deaths from alcohol-only and combined alcohol and opioid poisoning are smoother. Further, we have added T51.0 and T51.9 (toxic effect of alcohol) from contributing causes to form the final version (#3) of our alcohol poisoning definition which was used in all calculations.

Figure S1. Total number of deaths aged 18 or older in US 2000-19

from alcohol poisoning only, opioid poisoning only and alcohol and opioid poisoning

using three different versions of alcohol poisoning definition

Table S2. Number of deaths by three versions of alcohol poisoning definition (raw, adjusted and final) for alcohol poisoning only, opioid poisoning only and alcohol and opioid poisoning, for the total population, age 18 or older, and age 25 or older

| **Total** |  | Raw |  |  | Adjusted |  |  | Final |  |
| --- | --- | --- | --- | --- | --- | --- | --- | --- | --- |
| year | alc_only | opioid_only | alc_opioid | alc_only | opioid_only | alc_opioid | alc_only | opioid_only | alc_opioid |
| 2000 | 722 | 7598 | 792 | 3929 | 6800 | 1590 | 4154 | 6635 | 1755 |
| 2001 | 779 | 8698 | 785 | 3995 | 8103 | 1380 | 4259 | 7938 | 1545 |
| 2002 | 894 | 10987 | 917 | 4189 | 10237 | 1667 | 4458 | 10037 | 1867 |
| 2003 | 983 | 11939 | 990 | 4450 | 11123 | 1806 | 4718 | 10941 | 1988 |
| 2004 | 974 | 12757 | 986 | 4403 | 12064 | 1679 | 4694 | 11855 | 1888 |
| 2005 | 1010 | 13931 | 979 | 4807 | 13132 | 1778 | 5172 | 12863 | 2047 |
| 2006 | 1139 | 16457 | 1074 | 5162 | 15454 | 2077 | 5578 | 15114 | 2417 |
| 2007 | 4831 | 16508 | 1998 | 4840 | 16508 | 1998 | 5982 | 15993 | 2513 |
| 2008 | 5489 | 17047 | 2527 | 5489 | 17047 | 2527 | 6709 | 16588 | 2986 |
| 2009 | 5647 | 17963 | 2448 | 5647 | 17963 | 2448 | 6782 | 17511 | 2900 |
| 2010 | 5662 | 18646 | 2431 | 5662 | 18646 | 2431 | 6941 | 18168 | 2909 |
| 2011 | 5846 | 19877 | 2892 | 5846 | 19877 | 2892 | 7155 | 19484 | 3285 |
| 2012 | 5872 | 20103 | 3044 | 5872 | 20103 | 3044 | 7276 | 19635 | 3512 |
| 2013 | 6054 | 21671 | 3369 | 6054 | 21671 | 3369 | 7345 | 21171 | 3869 |
| 2014 | 6192 | 24761 | 3876 | 6192 | 24761 | 3876 | 7510 | 24230 | 4407 |
| 2015 | 6386 | 28594 | 4482 | 6386 | 28594 | 4482 | 7820 | 28024 | 5052 |
| 2016 | 6650 | 36560 | 5673 | 6650 | 36560 | 5673 | 8026 | 35786 | 6447 |
| 2017 | 6757 | 41201 | 6369 | 6757 | 41201 | 6369 | 8158 | 40510 | 7060 |
| 2018 | 6659 | 40463 | 6313 | 6659 | 40463 | 6313 | 8093 | 39831 | 6945 |
| 2019 | 6814 | 42526 | 7310 | 6814 | 42526 | 7310 | 8207 | 41922 | 7914 |
| **Age 18+** |  | Raw |  |  | Adjusted |  |  | Final |  |
| year | alc_only | opioid_only | alc_opioid | alc_only | opioid_only | alc_opioid | alc_only | opioid_only | alc_opioid |
| 2000 | 712 | 7532 | 790 | 3887 | 6738 | 1584 | 4112 | 6573 | 1749 |
| 2001 | 771 | 8583 | 785 | 3957 | 7990 | 1378 | 4221 | 7825 | 1543 |
| 2002 | 881 | 10868 | 915 | 4143 | 10120 | 1663 | 4412 | 9920 | 1863 |
| 2003 | 975 | 11799 | 987 | 4400 | 10987 | 1799 | 4666 | 10805 | 1981 |
| 2004 | 961 | 12574 | 981 | 4353 | 11885 | 1670 | 4642 | 11676 | 1879 |
| 2005 | 1004 | 13754 | 978 | 4752 | 12956 | 1776 | 5117 | 12688 | 2044 |
| 2006 | 1134 | 16239 | 1069 | 5114 | 15243 | 2065 | 5528 | 14904 | 2404 |
| 2007 | 4765 | 16267 | 1987 | 4774 | 16267 | 1987 | 5907 | 15758 | 2496 |
| 2008 | 5460 | 16819 | 2518 | 5460 | 16819 | 2518 | 6671 | 16364 | 2973 |
| 2009 | 5605 | 17760 | 2434 | 5605 | 17760 | 2434 | 6727 | 17309 | 2885 |
| 2010 | 5614 | 18425 | 2426 | 5614 | 18425 | 2426 | 6889 | 17950 | 2901 |
| 2011 | 5809 | 19662 | 2882 | 5809 | 19662 | 2882 | 7112 | 19270 | 3274 |
| 2012 | 5841 | 19956 | 3036 | 5841 | 19956 | 3036 | 7235 | 19488 | 3504 |
| 2013 | 6029 | 21515 | 3364 | 6029 | 21515 | 3364 | 7310 | 21016 | 3863 |
| 2014 | 6171 | 24610 | 3871 | 6171 | 24610 | 3871 | 7480 | 24079 | 4402 |
| 2015 | 6361 | 28423 | 4474 | 6361 | 28423 | 4474 | 7779 | 27853 | 5044 |
| 2016 | 6625 | 36361 | 5663 | 6625 | 36361 | 5663 | 7997 | 35589 | 6435 |
| 2017 | 6723 | 41050 | 6361 | 6723 | 41050 | 6361 | 8114 | 40360 | 7051 |
| 2018 | 6634 | 40293 | 6308 | 6634 | 40293 | 6308 | 8058 | 39662 | 6939 |
| 2019 | 6786 | 42320 | 7298 | 6786 | 42320 | 7298 | 8168 | 41719 | 7899 |
| **Age 25+** |  | Raw |  |  | Adjusted |  |  | Final |  |
| year | alc_only | opioid_only | alc_opioid | alc_only | opioid_only | alc_opioid | alc_only | opioid_only | alc_opioid |
| 2000 | 668 | 6904 | 734 | 3531 | 6152 | 1486 | 3747 | 5997 | 1641 |
| 2001 | 734 | 7779 | 729 | 3575 | 7213 | 1295 | 3830 | 7055 | 1453 |
| 2002 | 835 | 9858 | 833 | 3767 | 9159 | 1532 | 4019 | 8971 | 1720 |
| 2003 | 908 | 10596 | 900 | 3965 | 9836 | 1660 | 4217 | 9668 | 1828 |
| 2004 | 895 | 11200 | 900 | 3916 | 10562 | 1538 | 4194 | 10367 | 1733 |
| 2005 | 929 | 12304 | 876 | 4221 | 11576 | 1604 | 4571 | 11326 | 1854 |
| 2006 | 1048 | 14425 | 956 | 4580 | 13524 | 1857 | 4971 | 13203 | 2178 |
| 2007 | 4248 | 14459 | 1800 | 4255 | 14459 | 1800 | 5286 | 13986 | 2273 |
| 2008 | 4996 | 14919 | 2286 | 4996 | 14919 | 2286 | 6098 | 14484 | 2721 |
| 2009 | 5124 | 15902 | 2229 | 5124 | 15902 | 2229 | 6157 | 15474 | 2657 |
| 2010 | 5251 | 16437 | 2187 | 5251 | 16437 | 2187 | 6422 | 15986 | 2638 |
| 2011 | 5387 | 17528 | 2636 | 5387 | 17528 | 2636 | 6567 | 17155 | 3009 |
| 2012 | 5468 | 17914 | 2840 | 5468 | 17914 | 2840 | 6740 | 17468 | 3286 |
| 2013 | 5634 | 19367 | 3137 | 5634 | 19367 | 3137 | 6799 | 18887 | 3617 |
| 2014 | 5805 | 22236 | 3639 | 5805 | 22236 | 3639 | 7011 | 21723 | 4152 |
| 2015 | 6016 | 25712 | 4212 | 6016 | 25712 | 4212 | 7318 | 25164 | 4760 |
| 2016 | 6257 | 32797 | 5338 | 6257 | 32797 | 5338 | 7545 | 32056 | 6079 |
| 2017 | 6392 | 37377 | 6037 | 6392 | 37377 | 6037 | 7663 | 36717 | 6697 |
| 2018 | 6331 | 37085 | 6023 | 6331 | 37085 | 6023 | 7640 | 36485 | 6623 |
| 2019 | 6474 | 39109 | 6932 | 6474 | 39109 | 6932 | 7742 | 38544 | 7497 |

Table S3. Coefficient estimates of generalized least square (GLS) models predicting racial and ethnic and educational differences in US poisoning mortality rates (per 100,000) aged 18 and over 2000-2019 ^1^

|  | **Men** | | | **Women** | | |
| --- | --- | --- | --- | --- | --- | --- |
|  | **Alcohol-only** | **Opioid-only** | **Alcohol & Opioid** | **Alcohol-only** | **Opioid-only** | **Alcohol & Opioid** |
| Race and ethnicity effect at 2010 (ref. White) | | | | | | |
| Black | -1.072 (-1.319, -0.825)*** | -5.180 (-9.801, -0.559)* | -0.784 (-1.488, -0.080)* | -0.958 (-1.129, -0.786)*** | -4.095 (-7.652, -0.538)* | -0.460 (-0.678, -0.241)*** |
| Hispanic | -1.333 (-1.811, -0.855)*** | -5.697 (-11.418, 0.023) | -0.843 (-1.508, -0.178)* | -0.846 (-1.004, -0.687)*** | -8.118 (-13.780, -2.455)** | -0.399 (-0.555, -0.243)*** |
| Other | -0.156 (-0.497, 0.184) | -6.256 (-10.486, -2.027)** | -1.146 (-1.638, -0.655)*** | -0.500 (-0.668, -0.333)*** | -4.608 (-8.046, -1.170)** | -0.409 (-0.552, -0.265)*** |
| Education effect at 2010 (ref High education) | | | | | | |
| Low education | 5.216 (4.895, 5.536)*** | 18.457 (9.889, 27.025)*** | 2.312 (0.682, 3.943)** | 1.059 (0.842, 1.275)*** | 5.876 (4.081, 7.672)*** | 0.057 (-0.507, 0.621) |
| Medium education | 1.488 (1.300, 1.676)*** | 3.428 (1.387, 5.470)** | 0.683 (-0.466, 1.831) | 0.210 (0.018, 0.401)* | 1.793 (0.872, 2.714)*** | -0.157 (-0.682, 0.369) |
| Linear Year^2^ | 0.057 (0.044, 0.069)*** | 0.504 (0.077, 0.932)* | 0.087 (0.002, 0.172)* | 0.041 (0.021, 0.060)*** | 0.411 (0.016, 0.807)* | 0.082 (0.031, 0.133)** |
| Quadratic year^2^ | -0.003 (-0.006, -0.001)** | 0.013 (-0.010, 0.036) | 0.002 (-0.003, 0.007) | -0.002 (-0.005, 0.001) | 0.009 (-0.011, 0.029) | 0.002 (0.000, 0.005) |
| Race by linear year |  |  |  |  |  |  |
| Black x year | -0.050 (-0.078, -0.022)*** | -0.294 (-0.709, 0.121) | 0.027 (-0.043, 0.097) | -0.035 (-0.053, -0.018)*** | -0.343 (-0.732, 0.047) | -0.019 (-0.043, 0.005) |
| Hispanic x year | -0.058 (-0.110, -0.007)* | -0.693 (-1.142, -0.245)** | -0.095 (-0.158, -0.031)** | -0.053 (-0.072, -0.035)*** | -0.924 (-1.605, -0.243)** | -0.059 (-0.076, -0.041)*** |
| Other x year | -0.008 (-0.049, 0.034) | -0.686 (-1.112, -0.260)** | -0.145 (-0.198, -0.093)*** | -0.022 (-0.042, -0.002)* | -0.466 (-0.872, -0.060)* | -0.067 (-0.084, -0.050)*** |
| Race by quadratic year |  |  |  |  |  |  |
| Black x year sq. | 0.007 (0.002, 0.012)** | 0.020 (-0.010, 0.049) | 0.011 (0.003, 0.019)** | 0.003 (0.001, 0.006)* | -0.003 (-0.023, 0.018) | 0.002 (-0.001, 0.004) |
| Hispanic x year sq. | 0.005 (-0.002, 0.013) | -0.025 (-0.054, 0.004) | 0.000 (-0.007, 0.007) | 0.001 (-0.002, 0.004) | -0.037 (-0.071, -0.003)* | -0.003 (-0.005, 0.000)* |
| Other x year sq. | 0.003 (-0.003, 0.010) | -0.025 (-0.050, 0.000) | -0.005 (-0.011, 0.001) | 0.002 (-0.001, 0.006) | -0.015 (-0.036, 0.006) | -0.003 (-0.005, 0.000)* |
| Education by linear year |  |  |  |  |  |  |
| LowEd x year | 0.159 (0.124, 0.195)*** | 1.778 (1.256, 2.300)*** | 0.273 (0.145, 0.401)*** | 0.068 (0.045, 0.090)*** | 0.746 (0.568, 0.923)*** | 0.038 (-0.018, 0.093) |
| MedEd x year | 0.100 (0.079, 0.121)*** | 0.545 (0.332, 0.757)*** | 0.122 (0.022, 0.223)* | 0.046 (0.027, 0.065)*** | 0.238 (0.152, 0.324)*** | 0.002 (-0.047, 0.052) |
| Educ by quadratic year |  |  |  |  |  |  |
| LowEd x year sq. | 0.002 (-0.004, 0.008) | 0.105 (0.072, 0.138)*** | 0.024 (0.014, 0.035)*** | 0.003 (-0.001, 0.006) | 0.036 (0.022, 0.051)*** | 0.005 (0.002, 0.009)** |
| MedEd x year sq. | 0.000 (-0.004, 0.003) | 0.037 (0.016, 0.059)** | 0.009 (0.003, 0.015)** | 0.001 (-0.002, 0.004) | 0.011 (0.002, 0.020)* | 0.001 (-0.001, 0.004) |
| Intercept^3^ | 2.098 (1.989, 2.206)*** | 5.736 (1.463, 10.010)** | 1.121 (-0.176, 2.417) | 1.329 (1.135, 1.523)*** | 4.812 (1.459, 8.164)** | 0.847 (0.329, 1.365)** |

^1^ This table reports results replicating Table 1 analysis for age 18 and more. Model Predictors are linear and quadratic year centered at 2010, race and ethnicity and education dummies, and interaction between year, year square and race/ethnicity, and between year, year square, and education. N=240 for each model (20 years x 4 race groups x 3 education groups). All models allow for heteroskedastic variances and panel-specific first-order auto-regression (panel defined by race by education combination).

^2^ Linear and quadratic year effect for the reference group, i.e. white with high education

^3^ Intercept estimates average mortality rate for white with high education at year 2010

*p<.05, **p<.01, ***p<.001

Table S4. Coefficient estimates of generalized least square (GLS) models predicting racial and ethnic differences in educational inequalities in US poisoning mortality ratios calculated from mortality rates (per 100,000) aged 18 and more 2000-2019 ^1^

|  | **Alcohol-only** | | **Opioid-only** | | **Alcohol and Opioid** | |
| --- | --- | --- | --- | --- | --- | --- |
| **Men** | **Low Ed to High** | **Med Ed to High** | **Low Ed to High** | **Med Ed to High** | **Low Ed to High** | **Med Ed to High** |
| Race effect at 2010 (ref. White) | | | | |  |  |
| Black | 1.32 (0.79, 1.85)*** | 0.23 (0.03, 0.43)* | -0.35 (-1.57, 0.88) | 0.02 (-0.39, 0.43) | 1.73 (0.63, 2.83)** | 0.43 (0.04, 0.81)* |
| Hispanic | 0.53 (-0.03, 1.10) | 0.36 (0.04, 0.68)* | -2.40 (-3.51, -1.30)*** | -0.10 (-0.59, 0.39) | -1.26 (-2.15, -0.36)** | 0.43 (0.09, 0.76)* |
| Other | 8.47 (7.66, 9.27)*** | 2.92 (2.55, 3.30)*** | 1.56 (-0.50, 3.62) | 1.44 (0.34, 2.53)* | 9.77 (0.78, 18.75)* | 3.62 (1.35, 5.90)** |
| Year effect^2^ | 0.00 (-0.01, 0.01) | 0.03 (0.02, 0.04)*** | 0.23 (0.19, 0.27)*** | 0.09 (0.08, 0.10)*** | 0.03 (-0.06, 0.12) | 0.05 (0.03, 0.07)*** |
| Race by year |  |  |  |  |  |  |
| Black | 0.11 (0.01, 0.20)* | 0.03 (-0.01, 0.06) | 0.04 (-0.16, 0.24) | 0.02 (-0.05, 0.08) | 0.32 (0.13, 0.50)** | 0.10 (0.04, 0.17)** |
| Hispanic | -0.04 (-0.14, 0.06) | -0.01 (-0.07, 0.04) | -0.23 (-0.42, -0.04)* | -0.07 (-0.15, 0.02) | 0.06 (-0.09, 0.21) | 0.04 (-0.02, 0.10) |
| Other | 0.02 (-0.13, 0.16) | 0.08 (0.02, 0.15)* | -0.23 (-0.58, 0.12) | -0.07 (-0.26, 0.12) | -0.64 (-2.17, 0.89) | -0.30 (-0.70, 0.09) |
| Intercept^3^ | 3.68 (3.62, 3.74)*** | 1.75 (1.71, 1.78)*** | 7.34 (7.07, 7.60)*** | 2.91 (2.86, 2.96)*** | 6.76 (6.19, 7.32)*** | 2.57 (2.48, 2.67)*** |
| **Women** | **Low Ed to High** | **Med Ed to High** | **Low Ed to High** | **Med Ed to High** | **Low Ed to High** | **Med Ed to High** |
| Race effect at 2010 (ref. White) | | | | |  |  |
| Black | 0.99 (0.48, 1.50)*** | 0.03 (-0.22, 0.28) | -1.99 (-4.06, 0.08) | -0.69 (-1.08, -0.31)*** | 6.15 (2.82, 9.48)*** | 1.41 (0.24, 2.59)* |
| Hispanic | -1.02 (-1.30, -0.74)*** | -0.03 (-0.29, 0.22) | -4.28 (-5.47, -3.08)*** | -0.45 (-1.12, 0.22) | -2.51 (-3.25, -1.77)*** | -0.20 (-1.05, 0.65) |
| Other | 7.16 (5.32, 9.00)*** | 2.90 (1.85, 3.96)*** | -0.34 (-1.59, 0.91) | 1.45 (0.77, 2.13)*** | 2.41 (0.19, 4.64)* | 1.94 (0.90, 2.98)*** |
| Year effect^2^ | 0.03 (0.02, 0.05)*** | 0.04 (0.03, 0.05)*** | 0.43 (0.32, 0.55)*** | 0.15 (0.11, 0.18)*** | 0.17 (0.10, 0.25)*** | 0.09 (0.05, 0.12)*** |
| Race by year |  |  |  |  |  |  |
| Black | 0.00 (-0.09, 0.08) | -0.02 (-0.06, 0.02) | -0.11 (-0.39, 0.17) | -0.02 (-0.08, 0.04) | -0.29 (-0.86, 0.27) | -0.08 (-0.28, 0.12) |
| Hispanic | 0.06 (0.01, 0.11)* | 0.07 (0.02, 0.11)** | -0.50 (-0.68, -0.32)*** | -0.19 (-0.30, -0.07)** | -0.05 (-0.18, 0.07) | 0.02 (-0.13, 0.17) |
| Other | -0.49 (-0.81, -0.17)** | -0.12 (-0.30, 0.07) | -0.31 (-0.50, -0.13)** | -0.09 (-0.20, 0.02) | 0.19 (-0.20, 0.57) | 0.00 (-0.19, 0.18) |
| Intercept^3^ | 2.59 (2.52, 2.66)*** | 1.55 (1.50, 1.61)*** | 7.48 (6.58, 8.38)*** | 3.03 (2.81, 3.25)*** | 4.86 (4.40, 5.32)*** | 2.31 (2.09, 2.53)*** |

^1^ This table reports results replicating Table 2 analysis for age 18 and more. Model predictors are linear year centered at 2010, race and ethnicity and interaction between year and race and ethnicity. N=80 for each model (20 years x 4 race groups) except for models predicting alcohol and poisoning with N=79 for men and N=72 for women with missing years having zero mortality rate for high education. All models allow for heteroskedastic variances and panel-specific first-order auto-regression (panel defined by race and ethnicity). Model outcomes are low and medium education mortality rate divided by high education mortality rate, separately.

^2^ Linear year effect for the reference group, i.e. white

^3^ Intercept estimates average mortality education ratio for white at year 2010

*p<.05, **p<.01, ***p<.001

Table S5. Coefficient estimates of random-effect Poisson models predicting racial and ethnic and educational differences in US poisoning death counts aged 25 and more 2000-2019 ^1^

|  | **Men** | | | **Women** | | |
| --- | --- | --- | --- | --- | --- | --- |
|  | **Alcohol-only** | **Opioid-only** | **Alcohol & Opioid** | **Alcohol-only** | **Opioid-only** | **Alcohol & Opioid** |
| Race and ethnicity effect at 2010 (ref. White) | | | | | | |
| Black | -0.378 (-0.556, -0.200)*** | -0.880 (-0.979, -0.781)*** | -0.653 (-0.787, -0.518)*** | -0.807 (-0.909, -0.705)*** | -0.978 (-1.178, -0.778)*** | -1.008 (-1.241, -0.775)*** |
| Hispanic | -0.524 (-0.752, -0.296)*** | -1.038 (-1.262, -0.815)*** | -0.706 (-0.930, -0.483)*** | -0.896 (-1.277, -0.514)*** | -1.255 (-1.583, -0.926)*** | -1.130 (-1.735, -0.525)*** |
| Other | -0.222 (-0.683, 0.238) | -1.230 (-1.418, -1.042)*** | -0.990 (-1.276, -0.704)*** | -0.568 (-1.057, -0.080)* | -1.271 (-1.555, -0.987)*** | -1.138 (-1.520, -0.756)*** |
| Education effect at 2010 (ref High education) | | | | | | |
| Low education | 1.640 (1.290, 1.990)*** | 1.836 (1.640, 2.032)*** | 1.986 (1.776, 2.196)*** | 1.095 (0.634, 1.555)*** | 1.463 (1.136, 1.790)*** | 1.553 (1.213, 1.893)*** |
| Medium education | 0.830 (0.507, 1.152)*** | 1.131 (0.975, 1.287)*** | 1.145 (0.854, 1.436)*** | 0.565 (0.212, 0.917)** | 0.940 (0.695, 1.185)*** | 0.987 (0.617, 1.358)*** |
| Linear Year^2^ | 0.036 (0.033, 0.039)*** | 0.074 (0.072, 0.076)*** | 0.089 (0.081, 0.097)*** | 0.039 (0.034, 0.043)*** | 0.056 (0.051, 0.061)*** | 0.082 (0.076, 0.088)*** |
| Quadratic year^2^ | -0.002 (-0.002, -0.002)*** | 0.001 (0.000, 0.001)** | -0.001 (-0.001, 0.000)*** | -0.002 (-0.003, -0.002)*** | -0.003 (-0.004, -0.002)*** | -0.003 (-0.004, -0.002)*** |
| Race by linear year |  |  |  |  |  |  |
| Black x year | -0.006 (-0.011, 0.000) | -0.009 (-0.011, -0.007)*** | 0.009 (0.005, 0.013)*** | -0.007 (-0.011, -0.003)*** | -0.012 (-0.016, -0.008)*** | 0.008 (0.002, 0.015)* |
| Hispanic x year | -0.007 (-0.009, -0.006)*** | -0.043 (-0.047, -0.039)*** | -0.046 (-0.048, -0.044)*** | -0.021 (-0.037, -0.005)* | -0.042 (-0.054, -0.030)*** | -0.045 (-0.050, -0.041)*** |
| Other x year | -0.009 (-0.012, -0.006)*** | -0.027 (-0.034, -0.020)*** | -0.031 (-0.037, -0.025)*** | -0.018 (-0.032, -0.003)* | -0.031 (-0.041, -0.022)*** | -0.041 (-0.053, -0.028)*** |
| Race by quadratic year |  |  |  |  |  |  |
| Black x year sq. | 0.003 (0.003, 0.004)*** | 0.010 (0.009, 0.010)*** | 0.010 (0.010, 0.011)*** | 0.004 (0.002, 0.006)*** | 0.006 (0.004, 0.009)*** | 0.012 (0.010, 0.013)*** |
| Hispanic x year sq. | 0.001 (0.001, 0.002)*** | 0.006 (0.004, 0.007)*** | 0.007 (0.006, 0.008)*** | 0.002 (0.001, 0.003)** | 0.003 (0.002, 0.003)*** | 0.006 (0.004, 0.008)*** |
| Other x year sq. | 0.001 (0.000, 0.001)** | 0.001 (-0.001, 0.003) | 0.002 (0.000, 0.004)* | 0.002 (0.002, 0.003)*** | -0.001 (-0.002, 0.000)* | 0.001 (-0.001, 0.003) |
| Education by linear year |  |  |  |  |  |  |
| LowEd x year | -0.001 (-0.004, 0.002) | 0.035 (0.032, 0.037)*** | 0.007 (-0.001, 0.016) | 0.017 (0.011, 0.022)*** | 0.059 (0.054, 0.064)*** | 0.040 (0.033, 0.046)*** |
| MedEd x year | 0.016 (0.012, 0.019)*** | 0.034 (0.031, 0.036)*** | 0.022 (0.013, 0.030)*** | 0.029 (0.023, 0.035)*** | 0.048 (0.042, 0.054)*** | 0.042 (0.035, 0.048)*** |
| Educ by quadratic year |  |  |  |  |  |  |
| LowEd x year sq. | 0.001 (0.000, 0.001)*** | 0.001 (0.000, 0.001)* | 0.002 (0.002, 0.003)*** | 0.000 (0.000, 0.000) | 0.002 (0.001, 0.003)*** | 0.002 (0.001, 0.003)** |
| MedEd x year sq. | 0.000 (0.000, 0.000) | -0.001 (-0.001, 0.000) | 0.000 (0.000, 0.001) | 0.000 (-0.001, 0.000) | 0.001 (0.000, 0.002)* | 0.000 (-0.001, 0.002) |
| Intercept^3^ | -10.938 (-11.205, -10.671)*** | -10.329 (-10.460, -10.197)*** | -11.968 (-12.131, -11.805)*** | -11.537 (-11.797, -11.276)*** | -10.436 (-10.702, -10.170)*** | -12.704 (-12.921, -12.487)*** |

^1^ This table reports results as alternative to Table 1 analysis by fitting Poisson model predicting US poisoning mortality. Model Predictors are linear and quadratic year centered at 2010, race and ethnicity and education dummies, and interaction between year, year square and race/ethnicity, and between year, year square, and education. N=240 for each model (20 years x 4 race groups x 3 education groups). All models treat death count as outcome with population as offset. Robust standard errors were generated for Poisson model to adjust for potential over-dispersion.

^2^ Linear and quadratic year effect for the reference group, i.e. white with high education

^3^ Intercept estimates log mortality rate for white with high education at year 2010

*p<.05, **p<.01, ***p<.001

Figure S2. Age-standardized mortality rates for alcohol poisoning, opioid poisoning and combined alcohol and opioid poisoning for men and women by race and ethnicity categories from 2000 to 2019


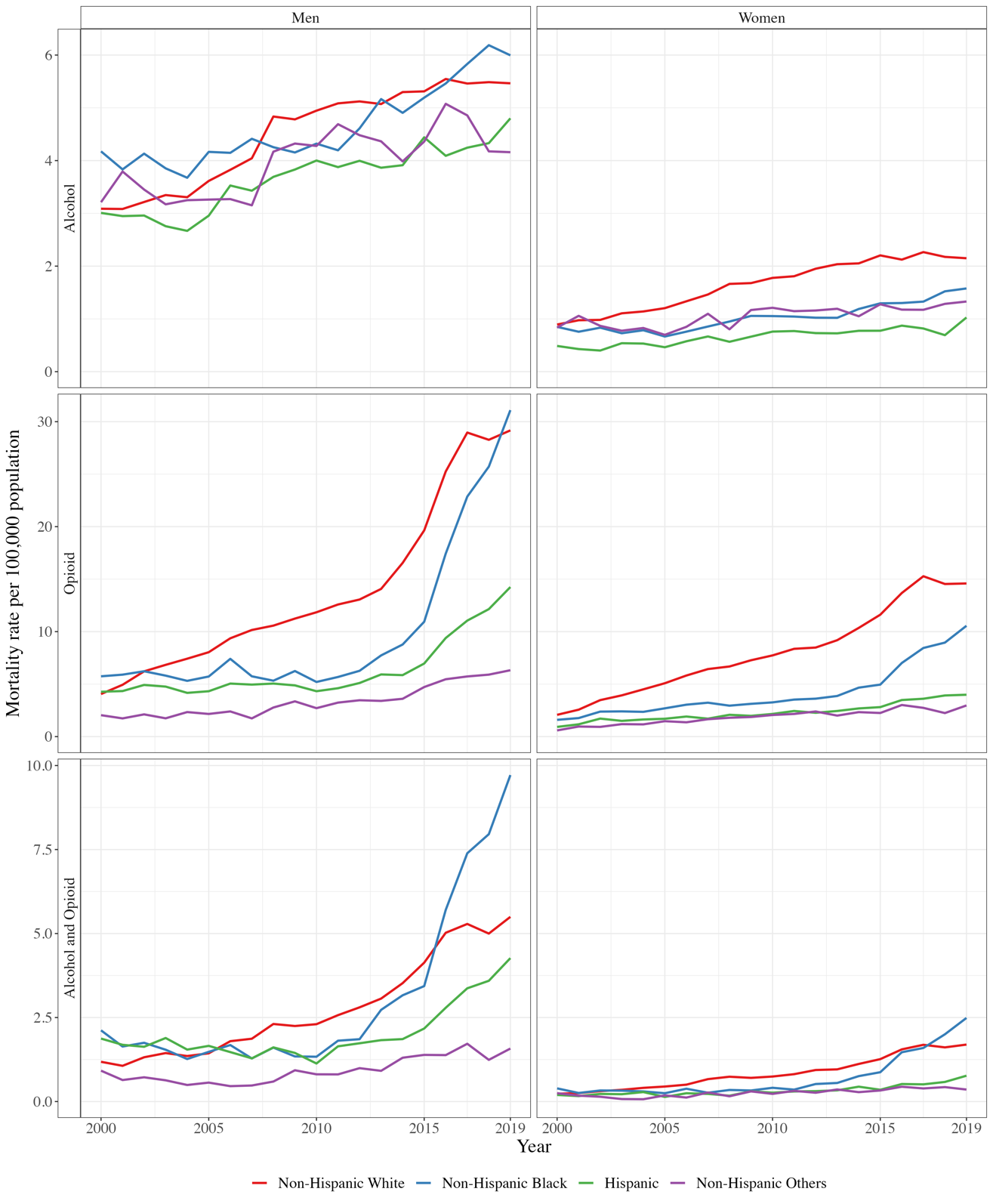


Note: *Alcohol poisoning only*: ICD-10 code X45 or F10.0 from underlying or contributing cause or T51.0 or T51.9 from contributing cause, and NOT opioid poisoning, i.e. both a) X40-X44, X60-X64, X85, Y10-Y14 from underlying cause and b) T40.0-T40.4, T40.6 from contributing cause. *Opioid poisoning only*: Both a) X40-X44, X60-X64, X85, Y10-Y14 from underlying cause and b) T40.0-T40.4, T40.6 from contributing cause, and NOT alcohol poisoning, i.e. X45 or F10.0 from underlying or contributing cause or T51.0 or T51.9 from contributing cause. *Alcohol and Opioid poisoning*: alcohol poisoning i.e. X45 or F10.0 from underlying or contributing cause or T51.0 or T51.9 from contributing cause, AND opioid poisoning, i.e. both a) X40-X44, X60-X64, X85, Y10-Y14 from underlying cause and b) T40.0-T40.4, T40.6 from contributing cause
